# Success Providing Postpartum Intrauterine Devices in Private-Sector Health Care Facilities in Nigeria: Factors Associated With Uptake

**DOI:** 10.9745/GHSP-D-16-00072

**Published:** 2016-06-20

**Authors:** George IE Eluwa, Ronke Atamewalen, Kingsley Odogwu, Babatunde Ahonsi

**Affiliations:** a Marie Stopes International Organization, Abuja, Nigeria; b United Nations Population Fund, Accra, Ghana

## Abstract

**Background:**

Use of modern contraceptive methods in Nigeria remained at 10% between 2008 and 2013 despite substantive investments in family planning services. Many women in their first postpartum year, in particular, have an unmet need for family planning. We evaluated use of postpartum intrauterine device (IUD) insertion and determined factors associated with its uptake in Nigeria.

**Methods:**

Data were collected between May 2014 and February 2015 from 11 private health care facilities in 6 southern Nigerian states. Women attending antenatal care in participating facilities were counseled on all available contraceptive methods including the postpartum IUD. Data were abstracted from participating facility records and evaluated using a cross-sectional analysis. Categorical variables were calculated as proportions while continuous variables were calculated as medians with the associated interquartile range (IQR). Multivariate logistic regression analysis was used to identify factors associated with uptake of the postpartum IUD while controlling for potential confounding factors, including age, educational attainment, marital status, parity, number of living children, and previous use of contraception.

**Results:**

During the study period, 728 women delivered in the 11 facilities. The median age was 28 years, and most women were educated (73% had completed at least the secondary level). The majority (96%) of the women reported they were married, and the median number of living children was 3 (IQR, 2–4). Uptake of the postpartum IUD was 41% (n = 300), with 8% (n = 25) of the acceptors experiencing expulsion of the IUD within 6 weeks post-insertion. After controlling for potential confounding factors, several characteristics were associated with greater likelihood of choosing the postpartum IUD, including lower education, having a higher number of living children, and being single. Women who had used contraceptives previously were less likely to choose the postpartum IUD than women who had not previously used contraception (adjusted odds ratio, 0.68; 95% confidence interval, 0.55 to 0.84).

**Conclusion:**

A high percentage (41%) of women delivering in private health care facilities in southern Nigeria accepted immediate postpartum IUD insertion. Scale-up of postpartum IUD services is a promising approach to increasing uptake of long-acting reversible contraceptives among women in Nigeria.

## Abstract (precis)

41% of women delivering in the social franchise private facilities chose the postpartum IUD. Factors associated with acceptance included lower education, higher parity, and being single. Scale-up of postpartum IUD services in both public and private facilities has the potential to significantly increase use of long-acting reversible contraception in Nigeria.

## INTRODUCTION

The postpartum period is a particularly vulnerable time for women for unintended pregnancy, with studies reporting pregnancy rates of 6% to 40% within the first postpartum year, depending on the population,^1^^-^^6^ and unmet need for modern contraceptives at 61%.^7^ Intrauterine devices (IUDs), including the TCu380A copper-bearing IUD and the levonorgestrel intrauterine system, confer similar contraceptive protection as that obtained with tubal ligation^7^^-^^9^ but are reversible and low-cost, thus making them an appealing contraceptive choice. Postpartum IUD insertion, in particular, is ideal for some women because the IUD does not interfere with breastfeeding; the timing is convenient for both women and the health care provider; and postpartum insertion is associated with less discomfort and fewer side effects than interval insertion (i.e., insertion of the IUD 6 weeks or more after delivery).^10^ In addition, postpartum IUD insertion affords a unique opportunity to address the contraceptive needs of women with limited access to medical care since the delivery and postpartum period may be one of the few times such women are in contact with medical services.^7^

Postpartum IUD insertion is ideal for some women for many reasons including convenient timing.

Safety of early postpartum IUD insertion has been studied since the 1960s^11^ and the procedure is practiced worldwide^7^^,^^12^; however, less is known about postpartum IUD uptake.^13^ Concerns over the possible risk of infection, bleeding, higher expulsion rates, and even provider reimbursements have limited use of the procedure. Barriers to uptake include cost, lack of provider knowledge or availability, 2-visit protocols for insertion, and misconceptions about IUDs.^11^^,^^13^^,^^15^^-^^17^

Nigeria is the seventh most populous country in the world; in 2015, the population was over 178 million people^17^ and the total fertility rate (TFR) was 5.5 children per woman.^18^ There has been minimal change in the TFR over the last 10 years, with TFR in 2003 reported at 5.7 children per woman.^18^ TFR is higher in rural areas (6.2) than in urban regions (4.7).^18^ The contraceptive prevalence rate (CPR) increased marginally between 2003 and 2013, from 13% to 15%.^18^ Only 10% of married women use a modern method of contraception, and use of long-acting reversible contraceptives (LARCs) is reported to be around 1%^18^ (0.2% for implants^19^ and 0.8% for IUDs^18^). The aim of this study was to determine key factors associated with uptake of postpartum IUD insertion in Nigeria. Such information is useful for informing, planning, and providing targeted programming for scale-up of LARCs in Nigeria.

## PROGRAM DESCRIPTION

Marie Stopes International Organization, Nigeria supports a social franchise network for private health care providers in the country to deliver quality family planning services at affordable cost. Health care providers within the network are trained on how to provide LARCs to clients in addition to a wide range of other methods. To address a gap in provision of postpartum family planning services, 11 health care providers within the network (1 provider in each of 11 facilities), who provided obstetric services but not postpartum family planning services, were preselected for competency-based training on postpartum IUD service provision.

To address a gap in postpartum family planning service provision in Nigeria, selected social franchise network providers received competency-based training in postpartum IUD services.

The competency-based training consisted of 2 days of didactic training and 3 days of practicum sessions both with models and with clients. Providers were required to competently insert 5 postpartum IUDs on the models before being allowed to do the insertions with clients under supervision.

In addition, all providers received routine (monthly) supportive supervision and mentoring after the training. Gynecologists who had been trained in postpartum IUD insertion conducted supportive supervision. Furthermore, to ensure quality postpartum IUD services were maintained during the study period, internal quality technical assurance (QTA) exercises were conducted twice a year (or more frequently for underperformers) by the Clinical Services Department of Marie Stopes International Organization, Nigeria, while external QTA exercises were conducted at least once a year by the Department of Obstetrics and Gynaecology, University of Benin Teaching Hospital, Edo State. Persistent underperformers were de-franchised from the network. QTA exercises focused on different thematic areas, including client focus, infection prevention, incidence reporting, medical emergency, technical competency (regarding insertion of postpartum IUDs), and supply management. After scoring providers in each thematic area, the auditors developed action plans for each provider in suboptimal thematic areas.

## MATERIALS AND METHODS

This study was conducted in South East and South South Nigeria among 11 private health care providers between May 2014 and February 2015. The states included Anambra, Abia, Delta, Ebonyi, Edo, and Enugu. All women who attended antenatal care at the 11 facilities were counseled on all available methods of contraception including the postpartum IUD. Women who presented at labor also received counseling about family planning methods including the postpartum IUD. Women who met medical eligibility criteria for IUD insertion and had no contraindications to IUD use were offered the postpartum IUD. Exclusion criteria included women with duration of ruptured membranes greater than 12 hours, prolonged labor, fever at presentation to the facility, postpartum hemorrhage, or signs of genital infections. All women offered postpartum IUDs provided written consent for the procedure. The women paid approximately US$5 for the procedure.

All providers used the TCu380A IUD (Pregna International Ltd., Mumbai, India), which they inserted either manually with their hands while the patient was still in the delivery room (post-placental insertion) or with Kelly’s forceps within 48 hours of delivery of the placenta according to the training manual. Given the large size of the postpartum uterus, providers were instructed to insert the IUD and the string completely into the uterus, with the IUD placed at the fundus of the uterus. In cases where the string was still visible, the string was tucked under the cervix.

Clients were taught how to check for the string to ensure the IUD had not been dislodged. All clients who accepted the postpartum IUD were given a 6-week postpartum follow-up appointment and were clinically assessed to determine if the IUD was still in place or had been expelled. Providers also checked for signs of infection at the follow-up visit. Clients were counseled to come back to the facility if they noticed an expulsion to have another IUD inserted, if willing, or to choose another method of contraception.

Demographic and reproductive health data were collected using a standardized client intake form. Data collected included educational attainment, parity, number of living children, and previous use of contraception. Data were extracted from the client intake forms and entered into Microsoft Excel. This was then imported into STATA 13.1 for statistical analysis.

Analysis included descriptive statistics with 95% confidence intervals (CIs) performed on quantitative data. Categorical variables were calculated as proportions, whereas continuous variables were calculated as medians with the associated interquartile range (IQR). Uptake of postpartum IUD was defined as the proportion of women who accepted the postpartum IUD among all those eligible for the method. Expulsion rate was defined as the proportion of women with an expelled IUD among those who had received the postpartum IUD. Chi-square test was used to test statistical significance between categorical variables, while the Wilcoxon rank-sum test was used for continuous variables. Bivariate logistic regression analysis was used to test associations between demographic and reproductive health variables, and uptake of postpartum IUD. Variables significant at *P*<_.20 were considered for inclusion in multivariate logistic regression models to identify factors independently associated with uptake of the postpartum IUD while controlling for potential confounders, including age, educational attainment, marital status, parity, number of living children, and previous use of contraception. Variables attaining significance at *P*<_.05 in the multivariate analysis were retained, based on the likelihood ratio test. The Nigerian Institute of Medical Research granted ethical approval for the study.

## RESULTS

### Background Characteristics

A total of 728 women delivered in the 11 facilities between May 2014 and February 2015. The median age was 28 years (IQR, 24–32 years). Most women had some level of education: 38% had achieved a tertiary-level education (post-secondary) and 35% had completed the secondary level. The large majority (96%) of the women were married. The median parity was 3 (IQR, 2–5), and the median number of living children was also 3 (IQR. 2–4). About one-third (36%) of the women had previously used contraception, all of whom were married.

### Postpartum IUD Uptake

Of the 728 women delivering during the study period, 41% (n = 300) of them accepted postpartum IUD insertion. About one-fourth (26%, n = 77) of the IUDs were inserted manually as immediate post-placental insertions, while the majority (74%, n = 223) were inserted within 48 hours of delivery with the use of forceps (Table 1). About 8% of women who chose the postpartum IUD (n = 25) experienced expulsion of the IUD, with the majority of the expulsions occurring among those inserted with forceps (72% with forceps vs. 18% manually; *P* = .78).

41% of women delivering during the study period accepted the postpartum IUD.

**TABLE 1:** Characteristics of Clients Delivering in the Social Franchise Clinics (N = 728), by Uptake of the Postpartum IUD, Southern Nigeria, May 2014–February 2015

	Accepted PPIUD (n = 300)	Rejected PPIUD (n = 428)	*P* Value
Age, years			
<25, No. (%)	50 (16.7)	112 (26.2)	
≥25, No. (%)	250 (83.3)	316 (73.8)	.002
Median (IQR)	29 (26, 34)	28 (24, 32)	<.001
Educational level, No. (%)			
None/primary	99 (33.0)	59 (13.8)	
Secondary	113 (37.7)	183 (42.8)	
Tertiary	88 (29.3)	186 (43.5)	<.001
Marital status, No. (%)			
Single	22 (7.3)	7 (1.6)	
Married	278 (92.7)	421 (98.4)	<.001
Parity			
0–1, No. (%)	8 (2.7)	101 (23.6)	
2–3, No. (%)	70 (23.3)	199 (46.5)	
4–5, No. (%)	149 (49.7)	98 (22.9)	
≥6, No. (%)	73 (24.3)	30 (7.0)	<.001
Median (IQR)	4 (3, 5)	3 (2, 4)	<.001
No. of living children			
0–1, No. (%)	9 (3.0)	102 (30.2)	
2–3, No. (%)	106 (35.3)	172 (50.9)	
4–5, No. (%)	151 (50.3)	58 (17.2)	
≥6, No. (%)	34 (11.3)	6 (1.8)	<.001
Median (IQR)	4 (3, 5)	2 (1, 3)	<.001
Previous use of contraception, No. (%)			
No	219 (73.0)	250 (58.4)	
Yes	81 (27.0)	178 (41.6)	<.001
Insertion technique, No. (%)			
Manual	77 (25.7)	NA	
Forceps	223 (74.3)	NA	
PPIUD expelled, No. (%)			
No	275 (91.7)	NA	
Yes	25 (8.3)	NA	

Abbreviations: IQR, interquartile range; NA, not applicable; PPIUD, postpartum intrauterine device.

*P* value derived from chi-square test for categorical variables and from Wilcoxon's rank-sum test for continuous variables.

### Factors Associated With Postpartum IUD Uptake

Table 2 outlines results from the multivariate logistic regression analysis of factors independently associated with uptake of the postpartum IUD. After controlling for potential confounding factors, women with no formal education or only a primary level of education were more likely to choose the postpartum IUD than women who had a tertiary level of education (adjusted odds ratio [AOR], 2.03; 95% CI, 1.20 to 3.42; *P* = .008). There was no difference in uptake between those with secondary level education and those with a tertiary level (AOR, 1.05; 95% CI, 0.68 to 1.64; *P* = .82). Women with parity 4–5 (AOR, 6.30; 95% CI, 1.36 to 28.72; *P* = .02) and ≥6 (AOR, 5.81; 95% CI, 1.15 to 29.27; *P* = .03) were more likely to accept the postpartum IUD than women with parity<_1. Compared with those who had 0–1 living child, those with 2–3 living children were more likely to choose the postpartum IUD (AOR, 4.56; 95% CI, 1.19 to 17.45; *P* = .03). The same was true for women with 4–5 living children (AOR, 8.30; 95% CI, 1.97 to 35.03; *P* = .004) and women with 6 or more living children (AOR, 17.76; 95% CI, 3.07 to 102.85; *P* = .001) when compared with women with 0–1 living child. Women who had used contraceptives previously were less likely than women who had not to choose the postpartum IUD (AOR, 0.68; 95% CI, 0.55 to 0.84; *P*<.001). Compared with married women, single women were more likely to choose the postpartum IUD (AOR, 6.76; 95% CI, 1.82 to 25.07; *P* = .004). Age was not associated with postpartum IUD uptake.

**TABLE 2:** Multivariate Analysis of Factors Associated With Postpartum IUD Uptake, Southern Nigeria, May 2014–February 2015

	Crude OR (95% CI)	Adjusted OR (95% CI)	*P* Value
Age, years			
<25	1	1	
≥25	1.77 (1.22, 2.57)	1.01 (0.61, 1.67)	.97
Educational level			
Tertiary	1	1	
Secondary	1.31 (0.92, 1.84)	1.05 (0.68, 1.64)	.82
None/primary	3.55 (2.35, 5.35)	2.03 (1.20, 3.42)	.008
Marital status			
Married	1	1	
Single	4.76 (2.01, 11.29)	6.76 (1.82, 25.07)	.004
Parity			
0–1	1	1	
2–3	4.44 (2.06, 9.59)	1.99 (0.48, 8.28)	.34
4–5	19.20 (8.94, 41.20)	6.30 (1.36, 28.72)	.02
≥6	30.72 (13.32, 70.88)	5.81 (1.15, 29.27)	.03
No. of living children			
0–1	1	1	
2–3	6.95 (3.39, 14.40)	4.56 (1.19, 17.45)	.03
4–5	29.51 (14.00, 62.20)	8.30 (1.97, 35.03)	.004
≥6	64.22 (21.30, 193.61)	17.76 (3.07, 102.85)	.001
Previous contraceptive use			
No	1	1	
Yes	0.72 (0.61, 0.85)	0.68 (0.55, 0.84)	<.001

Abbreviations: CI, confidence interval; IUD, intrauterine device; OR, odds ratio.

## DISCUSSION

The postpartum period is an ideal time to start contraception because motivation to adopt contraception at this time is high and it is convenient for both the client and the provider.^20^ Nearly two-thirds of women in their first postpartum year, however, have an unmet need for family planning.^21^

To the best of our knowledge, this is the first study to demonstrate the feasibility of providing postpartum IUD services among private health care providers and to determine factors associated with uptake of the postpartum IUD in Nigeria. We made several important observations. First and foremost, training providers in postpartum IUD counseling and insertion techniques resulted in a high acceptance rate: 41% of women delivering during the study period chose to use the postpartum IUD. Many women in Nigeria have limited access to health services, which may explain the high uptake of the postpartum IUD in this study. A study in Colombia reported that 95% of women who expressed the desire for immediate postpartum IUD insertion had it done compared with 45% of those who opted for delayed insertion,^7^ suggesting barriers to use when insertion is delayed. High uptake in our study may also be attributable to staff motivation, supportive supervision, and a manageable client-provider ratio in the private health facilities compared with public health facilities that are understaffed and demotivated with a client-provider ratio of 19 and 95 per 100,000 for doctors and nurses, respectively.^21^^,^^22^

Second, the rate of expulsion was low (8%) and consistent with or lower than expulsion rates reported in other published studies,^10^^,^^23^^-^^25^ indicating a high standard of insertion technique among the trained providers in this study. While expulsion rates with post-placental IUD insertion appear to be higher than with interval insertion, studies have concluded that immediate postpartum IUD insertion is safe and effective and the benefits outweigh the disadvantage of the increased risk of expulsion,^7^ particularly in settings with limited access to health services. It has also been suggested that expulsion rates are associated with the experience of the provider, irrespective of type of institutions and training levels.^12^^,^^26^^,^^27^ The 8% expulsion rate observed in our study indicates that 92% of clients who accepted the postpartum IUD continued on a highly effective method.

The expulsion rate of 8% in this study was consistent with or lower than rates reported in other published studies.

The third key observation of our study is that previous use of contraception does not necessarily translate to acceptability of the postpartum IUD; in fact, in our study women who had never previously used contraception were significantly more likely to accept postpartum IUD insertion than women who had used contraception in the past. Women who had used contraception in the past may have been comfortable with their previous method, and thus desired using that same method of contraception. However, a study among young Kenyan females^28^ showed no association between previous use of contraception and uptake of contraception. More research is needed to understand the association between previous use of contraception and uptake of the postpartum IUD.

Finally, several factors emerged as being independently associated with uptake of the postpartum IUD, including education, parity, number of living children, and marital status. These findings have salient implications for family planning programs in Nigeria. Women with primary or no formal education were more likely to use the postpartum IUD than women with tertiary-level education. This finding is in contrast with most studies in Nigeria that have shown that contraceptive use is higher among those with higher levels of education.^18^^,^^19^ However, those studies report analyses at univariate and bivariate levels while we report our findings using multivariate analysis. The higher likelihood of postpartum IUD use among those with lower education in our study may be related to the appeal of receiving contraception immediately after delivery, particularly since those with primary or no formal education were more likely to be of lower income status and therefore likely had more difficulty accessing health services. It may also be attributable to the fact that those with lower education had the highest parity and number of living children among the study group; our study also showed that the higher the number of living children or parity, the more likely the client would accept the postpartum IUD. Such women may be more motivated to limit childbearing than women with lower parity or number of living children. These findings are similar to that of other studies.^21^^,^^22^^,^^29^ Finally, our study showed that single women were 7 times more likely to accept the postpartum IUD than married women. While studies have shown more single women use contraceptives compared with married women, disaggregation of data usually shows the contraception methods most often used by single women are short-acting methods such as condoms and pills.^18^^,^^19^ Further studies are needed to understand the higher uptake of IUD use among single women in our study setting.

Women with lower education were more likely to use the postpartum IUD than those with higher education.

Single women were more likely to use the postpartum IUD than married women.

Despite substantial investments in family planning programs over the last 10 years, Nigeria’s modern contraceptive prevalence rate remains unchanged at 10% and only about 1% of this is for LARCs.^18^ Furthermore, unmet need is high at 20%.^19^ Postpartum IUD service delivery has the potential to address unmet need and increase contraceptive use among women in Nigeria and should be considered for scale-up in both public and private facilities.

Postpartum IUD service delivery has the potential to address unmet need and increase contraceptive use among women in Nigeria.

### Limitations

This study was cross-sectional in nature with the use of program data and thus should be interpreted with caution. Data on unwanted pregnancies and abortions among clients were not available; such factors may have influenced the uptake of contraception, especially among single women. While data on previous use of contraception were collected, the type of contraception was unknown and this may have influenced uptake of the postpartum IUD. Data on expulsion rates were collected over the first 6 weeks after insertion only, and thus our expulsion rates may be underreported. Lastly, this study was conducted exclusively among women who sought service from private facilities and suggests that they may be of higher socioeconomic status than women who would seek services from public services. While our results are promising, data on the socioeconomic status of clients were not available. The cost of US$5 for the postpartum IUD may be a barrier to uptake of this service among women attending public facilities and should be taken into consideration if this is to be implemented in public facilities.

## CONCLUSION

Our study documents the first time postpartum IUD contraception has been implemented in Nigeria on the scale reported here, demonstrating that postpartum IUD service delivery has the potential to make a significant contribution to much higher uptake of long-acting reversible contraception in Nigeria. Uptake of the postpartum IUD was 41%; lower education, high parity and number of living children, and being single were good predictors of women who accepted the method. With appropriate training, this procedure can be very successful given that expulsion of IUD after insertion was only 8%, indicating that 92% of acceptors would continue with a highly effective means of long-acting contraception when leaving the delivery facility.

## Notes

**Peer Reviewed**
